# D-Cbl Binding to Drk Leads to Dose-Dependent Down-Regulation of EGFR Signaling and Increases Receptor-Ligand Endocytosis

**DOI:** 10.1371/journal.pone.0017097

**Published:** 2011-02-14

**Authors:** Pei-Yu Wang, Li-Mei Pai

**Affiliations:** 1 Graduate Institute of Biomedical Science, Chang Gung University, Tao-Yuan, Taiwan; 2 Department of Biochemistry, Chang Gung University, Tao-Yuan, Taiwan; 3 Chang Gung Molecular Medicine Research Center, Chang Gung University, Tao-Yuan, Taiwan; University of Dayton, United States of America

## Abstract

Proper control of Epidermal Growth Factor Receptor (EGFR) signaling is critical for normal development and regulated cell behaviors. Abnormal EGFR signaling is associated with tumorigenic process of various cancers. Complicated feedback networks control EGFR signaling through ligand production, and internalization-mediated destruction of ligand-receptor complexes. Previously, we found that two isoforms of D-Cbl, D-CblS and D-CblL, regulate EGFR signaling through distinct mechanisms. While D-CblL plays a crucial role in dose-dependent down-regulation of EGFR signaling, D-CblS acts in normal restriction of EGFR signaling and does not display dosage effect. Here, we determined the underlying molecular mechanism, and found that Drk facilitates the dose-dependent regulation of EGFR signaling through binding to the proline-rich motif of D-CblL, PR. Furthermore, the RING finger domain of D-CblL is essential for promoting endocytosis of the ligand-receptor complex. Interestingly, a fusion protein of the two essential domains of D-CblL, RING- PR, is sufficient to down-regulate EGFR signal in a dose-dependent manner by promoting internalization of the ligand, Gurken. Besides, RING-SH2^Drk^, a fusion protein of the RING finger domain of D-Cbl and the SH2 domain of Drk, also effectively down-regulates EGFR signaling in *Drosophila* follicle cells, and suppresses the effects of constitutively activated EGFR. The RING-SH2^Drk^ suppresses EGFR signaling by promoting the endosomal trafficking of ligand-receptor complexes, suggesting that Drk plays a negative role in EGFR signaling by enhancing receptor endocytosis through cooperating with the RING domain of D-Cbl. Interfering the recruitment of signal transducer, Drk, to the receptor by the RING-SH2^Drk^ might further reduces EGFR signaling. The fusion proteins we developed may provide alternative strategies for therapy of cancers caused by hyper-activation of EGFR signaling.

## Introduction

Ubiquitination occurs via sequential activation and conjugation of ubiquitin to target proteins by ubiquitin activating enzyme (E1), ubiquitin-conjugating enzyme (E2) and ubiquitin ligase (E3) [1]. Aside from protein degradation, ubiquitination represents a crucial signal for the endocytosis of signaling molecules such as EGFR. The attenuation of EGFR signaling by endocytosis serves to properly control cell growth, differentiation, and normal developmental processes [2], [3], [4], [5]. Consistent with an intimate role in signaling regulation, as well as in other cellular processes, emerging evidence has shown that derailed endocytosis disrupts developmental processes and leads to cancer formation [6], [7].

A critical E3 ubiqutin ligase mediating the ubiquitiation-dependent receptor endocytosis is the proto-oncogene Casitas B-lineage lymphoma (Cbl), which was first identified as the cellular homolog of *v-cbl*, which induces pre-B-cell lymphomas and myeloid tumors [8], [9]. Cbl is involved in multiple signaling pathways, and plays a negative role in EGFR signaling that is conserved among many species [10]. In *Drosophila*, D-Cbl negatively regulates EGFR signaling in dorsoventral patterning during oogenesis [11], in eye development [12], [13], [14], and in border cell migration [15]. The Cbl recognizes receptor tyrosine kinase (RTK, such as EGFR) and non-receptor tyrosine kinases through a phosphotyrosine-binding (PTB) domain [10], [16]. Internalizing EGFR requires c-Cbl and Lys63-linked polyubiquitin chain modification mediated by c-Cbl is essential for sorting activated receptors to the lysosomal degradation compartment [17], [18], [19]. The RING finger domain in Cbl is highly conserved during evolution, particularly for critical amino acids related to E3 ligase activity [16], [20]. The C-terminal proline-rich (PR) domain for protein-protein interaction and the ubiquitin association (UBA) domain are found only in some members of the family [21]. In *Drosophila*, two major isoforms, D-CblL and D-CblS are generated from the single D-Cbl gene [22]; the shorter isoform D-CblS lacks the proline-rich and UBA domains.

Studies in mammals have shown that the endocytosis of EGFR involves multiple pathways, depending on EGF concentration and exhibits cell-based specificities [2], [23], [24]. Therefore, redundancy of multiple pathway of endocytosis made it difficult to dissect which molecule the process requires. The *Drosophila* eggshell patterning has served as a sensitive and simple system to read out the levels of EGFR signaling levels [25], [26], thus representing an ideal model of mechanistic studies. The advantage of this in vivo system is that it provides physiological conditions with a gradient of ligand concentration to induce different levels of EGFR activation that is reflecting through the D/V patterning of eggshell and embryo. The Gurken, a TGF-α homolog, is produced by the oocyte and activates EGFR in follicle cells to specify the dorsal cell fates, followed by attenuation of EGFR signaling via negative regulators, such as *sprouty*, and *kekkon*, which together determine the area of the follicle epithelium where the dorsal appendages (DAs) form [27], [28]. Importantly, examining the Gurken distribution in loss of D-Cbl and D-CblL over-expression conditions has revealed that D-CblL promotes endocytosis of ligand-receptor to control the amount of available ligand. We demonstrated that D-CblL facilitates the activated receptor to traffic through the endocytic pathway for terminating signaling at lysosomal degradation compartment [29]. Therefore, over-expression of D-CblL at different levels resulted in different degrees of ventralization, corresponding to phenotypes resulting from different severities of *gurken* mutant alleles [30]. This dose-dependent, negative effect on EGFR signaling is specific to D-CblL and is not produced by over-expression of D-CblS.

To understand how D-CblL controls EGFR signaling at the molecular level, this study investigates which molecular interaction with D-CblL is sufficient to facilitate the endocytosis of the ligand-EGFR complex in *Drosophila* egg chambers. This work first demonstrates that the factor Downstream of receptor kinase (Drk) plays a major role in D-CblL mediated down-regulation of EGFR signaling in a dose-dependent fashion. In addition, E3 ligase activity is required for D-CblL activity, because over-expression of Δ70Z-D-CblL, an E3 defective mutant, blocked ligand-receptor internalization and produced a dominant-negative effect. We generated the RING-SH2^Drk^ chimeric protein, containing two functional domains of D-Cbl and Drk, and found that this chimeric protein not only attenuated EGFR signaling, but also down-regulated constitutively activated EGFR, λ-top. We further demonstrated that RING-SH2^Drk^ suppresses EGFR signaling by enhancing the endosomal trafficking of the ligand-receptor complex and interfering with the recruitment of the endogenous Drk.

## Results

### Drk plays a major role in D-CblL mediated down-regulation of EGFR signaling

In this study, we set out to elucidate the molecular mechanism by which D-CblL promotes the endocytosis of the ligand-receptor complex. Since this effect of D-CblL was not observed for D-CblS even when expressed at a similar level [30], we suspected that D-CblL may mediate the internalization by its extra C-terminus that is distinct from D-CblS. In mammals, Grb2 (Growth factor receptor binding protein 2), Eps15 and the CIN85-Endophilin complex are involved in Cbl-mediated down-regulation of EGFR signaling [31], [32], [33]. We then tested for their involvement in D-CblL mediated down-regulation of EGFR signaling in *Drosophila* oogenesis by a sensitive genetic assay. While *Drosophila Eps15*, *endophilin A* and *endophilin B* had no or minor effects (Table S1), the *Drosophila* Grb2 homolog *drk* exhibits a strong link to D-CblL activity described below.

**Figure 1:**
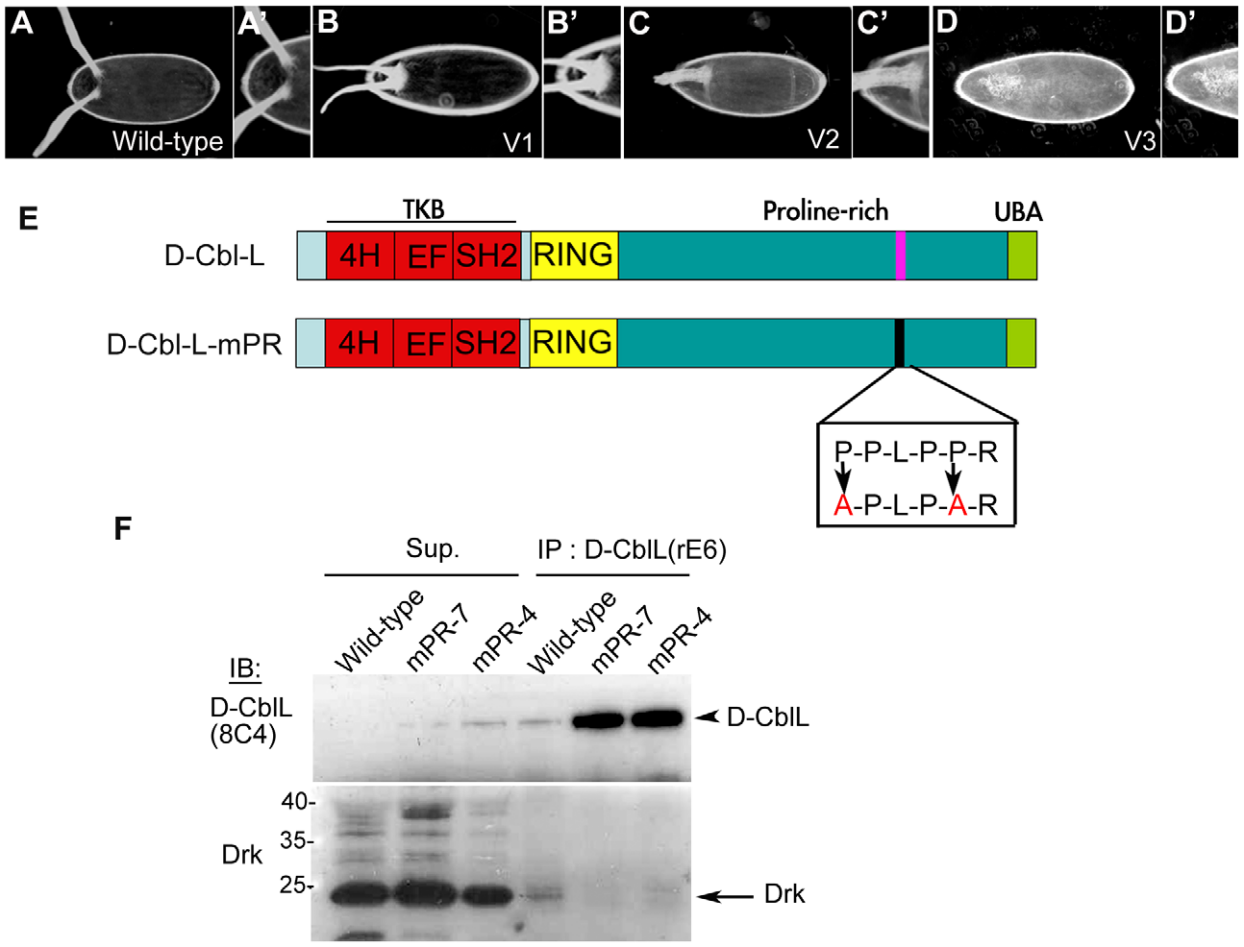
D-CblL directly interacts with Drk through its PR motif. (A and A′) The EGFR activity is correlated with the morphology of dorsal appendages. Two dorsal appendages indicate normal activity of EGFR (wt). (B and B′) Two dorsal appendages fused at the base indicate low level of EGFR activity (V1), whereas (C and C′) one fused dorsal appendage indicates a even lower level of EGFR activity (V2). (D and D′) No appendage indicates the lowest level of EGFR activity (V3). (E) The schematic structure shows the wild-type D-CblL, which contains the TKB domain in its N-terminus (red), the RING finger domain (yellow) and the proline-rich domain in the C-terminus. The predicated binding motif of Drk on D-CblL is PPLPPR (shown in purple). The first and fifth prolines of the PR motif on D-CblL-mPR mutant were replaced by alanines (shown in Box). (F) The D-CblL was immunoprecipitated from *Ore*R (wild-type) or two *hs83-D-cblL-mPR; cbl^F165^* rescued fly lines (line 4 and line 7) by the anti-D-CblL antibody (rE6). The immunoprecipitates (IP-10 µl out of 30 µl) were separated by the SDS-PAGE, and detected by the anti-D-CblL antibody (8C4) and the anti-Drk antibody. Supernatants (Sup.) were produced after immunoprecipitating, indicating the ability of immunoprecipitation in this experiment. 30 µl out of 400 µl total lysate was analyzed by western blotting in each lane. The Drk signal is near 24KD (arrow).

Mammalian studies demonstrated that c-Cbl is recruited to EGFR through directly binding the Y1045 residue of EGFR or indirectly interacting with Grb2 [34], [35], [36], [37]. The Tyr1068/1086 of EGFR is the direct docking site for the SH2 domain of Grb2 [38]. The SH3 domain of Grb2 binds to the proline-rich region of D-CblL, which is absent in D-CblS. We used the *drk^EOA^* mutant, which loses binding to EGFR caused by mutation in the SH2 domain [39], for a genetic interaction assay. The ventralized effect by *EQ1-Gal4*-driven over-expression of D-CblL in the follicle cells was significantly reduced in the heterozygous *drk^EOA^* mutant background (Table 1 and Figure 1A–D). *EQ1-Gal4* is mainly expressed in the follicle cells [30]. We reasoned that if Drk was required for the effect of D-CblL over-expression, the interruption of interaction between Drk and D-CblL would block the D-CblL over-expression effects. To address this issue, we found one consensus sequence PPLPPR of the Grb2/Drk binding motif on D-CblL (named PR), and generated a mutant in this motif by replacing the first and fifth prolines with alanines (mPR) (Figure 1E). The results from the yeast two hybrid system showed that the wild-type PR, but not the mPR, interacted with full-length Drk (Figure S2). Consistently, in the anti-D-Cbl immunoprecipitation assay, much less Drk was pulled down in the D-CblL-mPR complex than in the wild-type D-CblL complex, even though D-CblL-mPR was expressed at a higher level compared to that of D-CblL (Figure 1F). We then over-expressed D-CblL-mPR or D-CblL in follicle cells using *EQ1-Gal4*. At comparable levels (Figure S1A), the over-expression effects of D-CblL-mPR on EGFR signaling were much weaker than that of D-CblL. Furthermore, the effect of D-CblL-mPR over-expression was not suppressed in the heterozygous *drk^EOA^* mutant background, suggesting that PR of D-CblL might be a critical binding domain for Drk (Table 1).

**Table 1:** The genetic interaction between D-CblL and Drk depends on the PR^638-643^ motif of D-CblL.

At 25°C	% Eggshell phenotype*				
Progenies#	V3¥	V2¥	V1¥	Wt¥	N
*drk^EOA^/+*	0	0	3	97	421
*L-A10*	1	40	38	21	294
*L-A10* in *drk^EOA^/+*	0	29	56	15	247
*L-A12*	5.2	66.2	26.3	2.3	311
*L-A12* in *drk^EOA^/+*	0	27	63	10	176
*mPR-AE*	0	0	25	75	212
*mPR-AE* in *drk^EOA^/+*	0	0	25	75	390
*mPR-GB*	0	47	49	4	250
*mPR-GB* in *drk^EOA^/+*	0	50	50	0	362

*The number shows percentage in each phenotype.

¥ The Egfr activity is correlated with morphology of dorsal appendage, two dorsal appendages indicate normal activity of Egfr (Wt), two dorsal appendages fused at the base indicate low level of Egfr activity (V1), one fused dorsal appendage indicates the lower level of Egfr activity (V2), and no appendage indicates the lowest level of Egfr activity (V3).

# *EQ1-Gal4*-driven over-expression of each D-CblL or D-CblL-mPR transgenic line was ether in the wild-type background or in the heterozygous *drk^EOA^* mutant background.

We further analyzed the function of the D-CblL-mPR mutant using a constitutive expression promoter, HS83 [40], which was also used in rescue assays by D-CblL and D-CblS. Two transgenic lines, *hs83-D-cblL-mPR-7* and *hs83-D-cblL-mPR-4*, rescued the lethality of the *cbl^F165^* null mutant and 75%∼80% of the expected animals developed to adults (Table 2). The rescue ability of the D-CblL-mPR mutant was comparable to that of D-CblS (90∼100%). Moreover, unlike the wild-type D-CblL, *cbl* mutant flies rescued by D-CblL-mPR showed no pattern defect in wing or the eggs they laid (data not shown), although the expression level of *hs83-D-cblL-mPR* was similar to that of *hs83-D-cblL* (Figure S1B). These data indicate that the interaction between D-CblL and Drk underlies the functional difference between the D-CblS and D-CblL, and provide the basis for the dose-dependent, negative effects of D-CblL on EGFR signaling. However, this interaction is not essential for D-CblL function in terms of the normal restriction of EGFR signaling.

**Table 2:** *Hs83-D-CblL-mPR* rescues the lethality of *D-cbl^F165^* mutant.

	*Hs83-D-cbl* gene			
	2 copies	1 copy		
Line¶	Rescued ability (%)	Rescued ability (%)	V2* (%)	N
S-2	100	90	0	368
L-6	0	12	97	66
L-mPR-7	80	n.d¥	0	712
L-mPR-4§	-	75	0	443

¶ Each transgenic line was tested at 25°C.

*V2 phenotype indicated V2 phenotype, which is one fused dorsal appendage.

¥ n.d.: non-detected

§ This transgenic line is homozygous lethal.

### The E3 ligase activity of D-CblL is essential for its negative role in EGFR signaling

**Figure 2:**
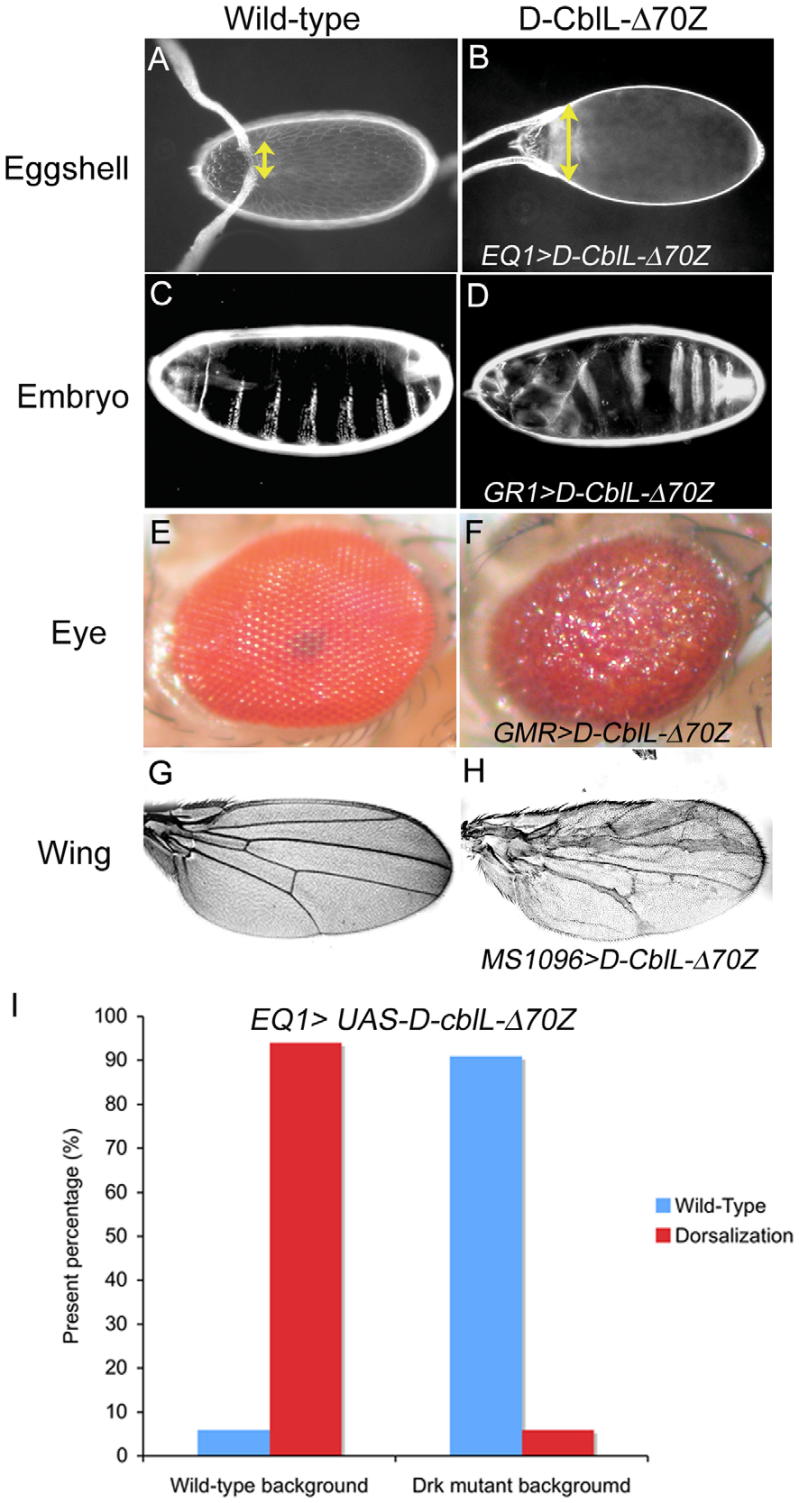
Dominant negative effects of D-CblL-Δ70Z. (A) This picture is a dorsal view of the wild-type eggshell. The dorsal midline is the region between the two dorsal appendages (indicated by yellow arrow). (B) Expression of D-CblL-Δ70Z in follicle cells driven by *EQ1-Gal4* resulted in a dorsalized eggshell characterized by an expanded dorsal midline (yellow arrow). (C) In the wild-type embryo, the eight ventral denticle belts represent the ventral structure. (D) Expression of D-CblL-Δ70Z in the follicle cells driven by *GR1-Gal4* resulted in a dorsalized embryo that lost the anterior ventral structure. (E) Wild-type adult eye. (F) Ubiquitous expression of D-CblL-Δ70Z in eyes driven by *GMR-Gal4* generated a rough eye phenotype. (G) Wild-type adult wing. (H) Expression of D-CblL-Δ70Z in the wing by *MS1096-Gal4* caused an extra vein phenotype. (I) The effect of *UAS-D-cblL-Δ70Z* expression could be suppressed in *drk^EOA^/+* background. The percentage of the wild-type and dorsalized eggshell are indicated in the blue and red column, respectively.

A screen for D-Cbl loss-of-function alleles has identified mutations in the RING domain [13]. Indeed, mouse fibroblasts that express Δ70Z-Cbl, an E3 defective mutant with a deletion of 17 amino acids prior to the RING finger domain, show increased EGFR activation upon ligand stimulation [41], [42]. In addition, the *D-v-cbl* and *D-cblS-onco* (D-CblS-Δ70Z) act as dominant negative mutants in the *Drosophila* eye and wing, which is presumably resulted from competing with wild-type D-Cbl for binding to the EGFR [43]. However, one proposed inhibitory function of Cbl in EGFR signaling is acting as an competitor with Sos (Son of sevenless) by binding to Grb2, thereby blocking signaling through the Ras-MAPK pathway [21]. We decided to test whether the E3 activity is essential for D-CblL function in the dose-dependent EGFR regulation. First we tested the involvement of ubiquitination in D-CblL-mediated regulation using the E2-conjugase mutant, *eff^8^*, which has been shown to be involved in *D-v-cbl* function [43], [44], [45]. The effect of D-CblL over-expression was reduced in the *eff^8^* heterozygous mutant (Table S2), indicating the attribution of ubiquitination in D-CblL effects. A deletion mutant similar to D-CblS-Δ70Z, which should be E3 defective, was generated for D-CblL, and notably dominant negative effects on EGFR signaling were observed upon its over-expression in the wing, eye and follicle cells (Figure 2). Significantly, the dominant effect of D-CblL-Δ70Z could be suppressed in *drk^EOA^* heterozygous mutant background (Figure 2I), indicating that the interaction between Drk and D-CblL is required for the dose-dependent effect of D-CblL on EGFR signaling. Furthermore, the *HS83-D-CblL-Δ70Z* could not rescue the *cbl^F165^* null mutant (data not shown), reinforcing the dependence of the negative role of D-CblL in EGFR signaling depends on the E3 activity.

**Figure 3:**
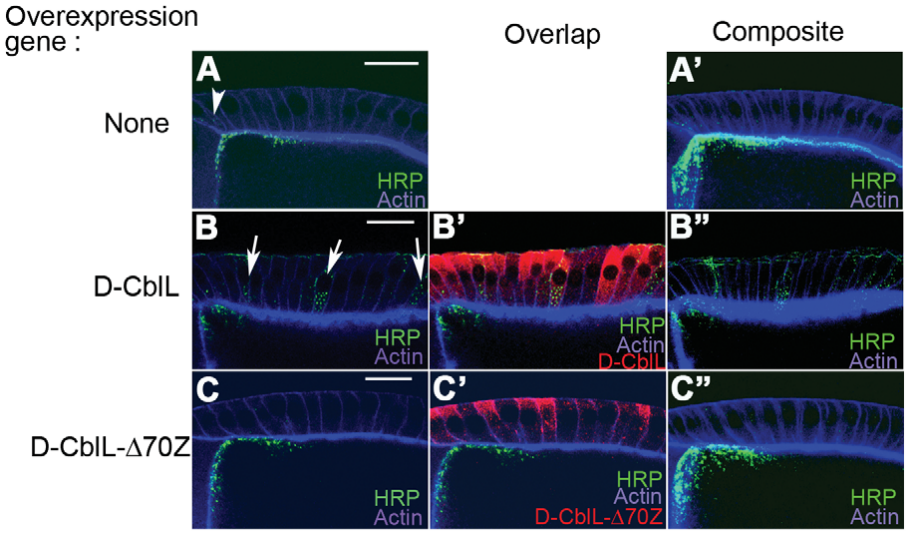
Expression of the D-CblL-Δ70Z mutant reduces endocytosis of Grk-receptor in follicle cells. (A and A′) The HRP signals represented the ligand-receptor complex in the follicle cell (green), and were detected in the dorsal-anterior corner of the middle stage egg chambers (arrowhead). (B, B′ and B″) Over-expression of D-CblL (red) promoted endocytosis of the ligand-receptor in follicle cells (arrows). (C, C′ and C″) However, expression of D-CblL-Δ70Z (red) significantly reduced HRP-Grk signals in the follicle cells. (B′ and C′) The D-CblL or D-CblL-Δ70Z expression are shown in B′ and C′, respectively. The composite image of several confocal sections of an egg chamber with HRP-Grk. Scale bars: 20 µm.

We had previously shown that there is an increase of the endocytic Gurken (HRP-Gurken) in follicle cells after D-CblL over-expression, implying that D-CblL promotes the endocytosis of the Gurken-EGFR complex [29]. To clarify the requirement of ubiquitination in D-CblL-mediated ligand-receptor endocytosis, the distribution of HRP-Gurken in follicle cells was examined. The HRP-Gurken signal was abolished in follicle cells with D-CblL-Δ70Z over-expression (Figure 3C, C′ and C″), whereas the signal was clearly detected in wild type or D-CblL expressing follicle cells (Figure 3 A and B). Taken together, our results showed that the E3 activity of D-CblL plays an essential role in promoting ligand-receptor endocytosis.

### The D-CblL fusion proteins down-regulate EGFR signaling

**Figure 4:**
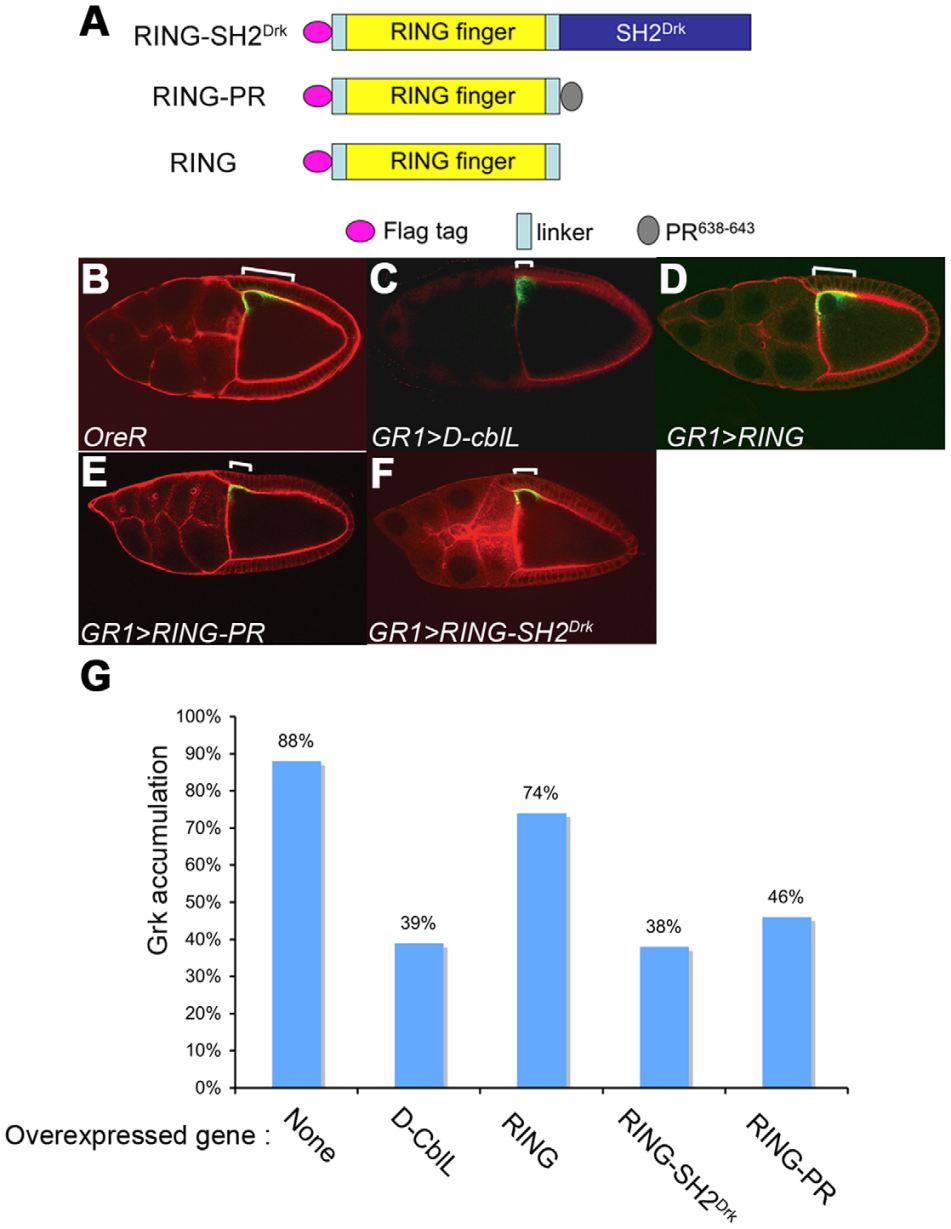
The RING domain fusion protein alteres the Grk distribution. (A) The schematic structure shows the constructions of E3 fusion proteins. RING-SH2^Drk^, the RING finger domain of D-CblL (red orthogon) fused with the SH2 domain of Drk (yellow orthogon). RING-PR, the RING finger domain was fused with PR motif (amino acid 638 to 643; PPLPPR; gray orthogon). RING was composed with the RING finger domain and the linker (blue orthogon) of D-CblL. The RING, RING-PR and RING-SH2^Drk^ were in-frame fused with a Flag-tag (red circle). (B-F) Gurken is asymmetrically expressed (green) in the dorsal-anterior corner of the middle-stage wild-type egg chamber (B). The normal Gurken expression pattern spreads about 8 to 10 follicle cells (indicated by white line in B). Expression of D-CblL (C), RING-PR (E), or RING-SH2^Drk^ (F) led to a reduced Grk distribution (white lines shown in C, E, F), but no significant effect was observed in the egg chamber expressing RING (D). (G) Quantitative data of B, C, D, E, and F, and the percentages indicated middle stage egg chambers with a normal pattern (*p<0.05; ** *p*<0.001).

Based on the functional implications of the D-CblL's interaction with Drk and E3 ligase activity, we therefore aimed to test next whether these two functional domains are sufficient to effectively down-regulate EGFR. A fusion protein containing the RING finger domain and the PR motif of D-CblL was generated (Figure 4 A). We expected that the fusion protein could interact with EGFR through binding to Drk. In line with this notion, we also generated a chimeric protein that contained the RING finger domain of D-CblL and the SH2 domain of Drk (Figure 4 A). This RING-SH2^Drk^ chimera should be able to bind to the activated EGFR on pY1068 or pY1086 that can be recognized by the SH2 domain of Drk.

To test the effects of these fusion proteins on EGFR signaling, ectopic expression of UAS-Flag-RING-SH2^Drk^ and of UAS-RING-PR were induced in follicle cells by *GR1-Gal4* at 29°C and 32°C, respectively (Figure S1). At 25°C, only low levels of D-Cbl fusion proteins were expressed, resulting in a slight defect on the eggshell morphology (data not shown). High-level expression of RING-SH2^Drk^ at 29°C resulted in significantly reduced EGFR signaling, and about half of the eggshells showed an intermediate ventralization phenotype indicated by the fusion of two dorsal appendages (Table 3 and Figure 1C). The effects were correlated with expression levels of the fusion protein (Figure S1). Even though expression of RING-PR had a weaker effect than that caused by expression of RING-SH2^Drk^, high level expression of RING-PR, induced by hsGal4 (Figure S1D), also effectively down-regulated EGFR signaling and caused about 1/3 of the eggshells to display the intermediate ventralization phenotype (Table 3, Figure 1C). Consistent with our previous observations [29], Gurken distribution outside of the follicle cells was also reduced when RING-SH2^Drk^ or RING-PR were over-expressed in follicle cells, similar to the effects caused by D-CblL over-expression (Figure 4). These results showed that these small fusion proteins, as well as full length D-CblL, could down-regulate EGFR signaling in a dose-dependent manner. Furthermore, RING-SH2^Drk^ and RING-PR can promote the internalization of ligand-receptor complexes and lead to a reduction of extracellular Gurken distribution.

**Table 3:** RING-PR and RING-SH2^Drk^ down-regulate Egfr signaling in a dose dependent manner.

	% Eggshell phenotype				
Genotypes	V3	V2	V1	Wt	N
**At 25°C**					
* flag-D-CblL-1*	18	80	1	1	364
* flag-D-CblL-6*	4	93	3	0	243
**At 29°C (32°C)**					
* RING-A* ¶	0 (0)	0 (3)	1 (12)	99 (85)	271 (367)
* RING-B* ¶	0 (0)	0 (5)	0(6)	100 (89)	233 (333)
* RING-B2; RING-B* ¥	0	1	3	96	148
* RING-SH2^Drk^-2* ¶	0 (0)	2 (40)	26 (48)	72 (12)	334 (503)
* RING-SH2^Drk^-5* ¶	0 (0)	19 (58)	24 (36)	57 (6)	376 (430)
* RING-PR-A* ¶	0 (0)	8 (2)	25 (22)	67 (76)	220 (374)
* RING-PR-D* ¶	0 (0)	0 (0)	12 (7)	88 (93)	154 (164)
* RING-PR-A; RING-PR-D* ¥	0	34	23	43	253

§ The egg collection was done at 25°C, and expressions were driven by *EQ1-Gal4*.

¶ The egg collection was done at two temperatures, 29°C and 32°C, and expressions were driven by *GR1-Gal4*.

¥ Two copies of transgene driven by *hsGal4* were induced at 37°C for 1 hour, and then eggs were collected at 25°C.

### RING-SH2^Drk^ down-regulates EGFR signaling through endosomal sorting and competition with Drk

**Figure 5:**
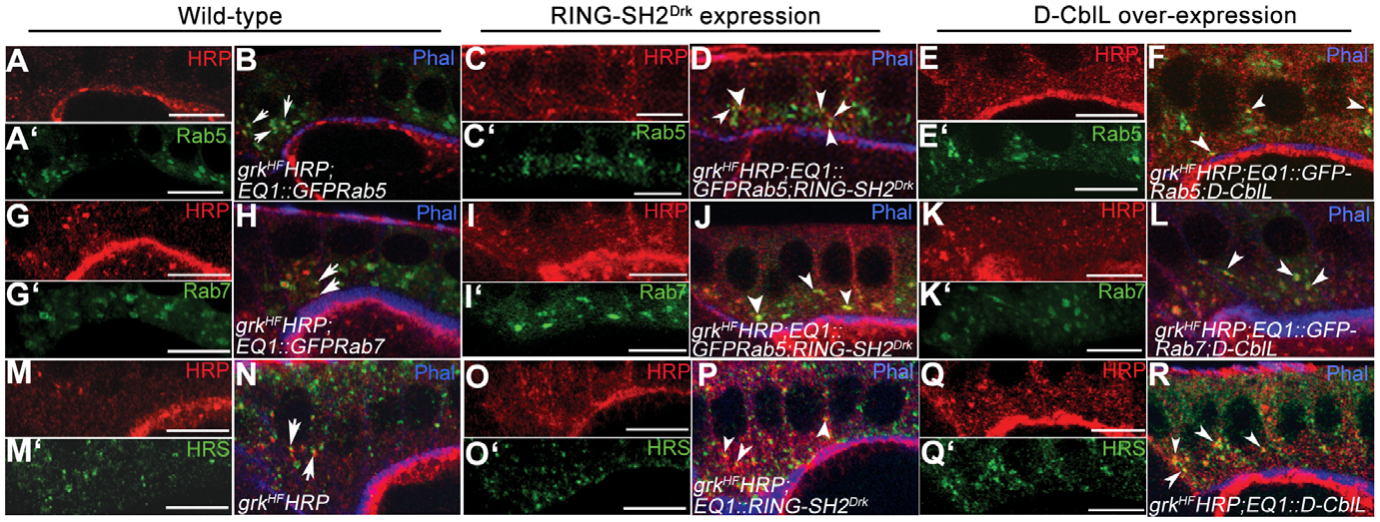
Expression of D-CblL and RING-SH2^Drk^ promotes the lysosomal sorting of the Grk-receptor complex. (A–F) Some HRP signals were colocalized with Rab5-GFP signals in wild-type follicle cells (A, A′ and arrow in B), and more colocalized signals of HRP and Rab5 were detected in follicle cells expressing RING-SH2^Drk^ (C, C′ and arrowhead in D), and in cells expressing D-CblL (E, E′ and arrowhead in F). (G–L) Similarly, significantly more colocalized signals were detected in cells expressing RING-SH2^Drk^ (I, I′ and arrowhead J) and D-CblL (K, K′ and arrowhead in L) than that detected in wild-type follicle cells (G, G′ and arrow in H). (M–R) The colocalization signal of HRP and Hrs were shown in the wild-type (M, M′ and arrow in N), or in cells expressing RING-SH2^Drk^ (O, O′ and arrowhead in P), and in cells expressing D-CblL (Q, Q′ and arrowhead in R).

We previously demonstrated that D-CblL promotes the internalization of the Grk/EGFR complex via the Rab5/Rab7 endocytic pathway [29]. To determine the route in endosomal trafficking of RING-SH2^Drk^-mediated endocytosis, we assayed the HRP-Grk/EGFR complex using the anti-HRP antibody in follicle cells expressing the RING-SH2^Drk^ chimera protein. More HRP-Grk signals were co-localized with Rab5-GFP and Rab7-GFP in follicle cells expressing either D-CblL (Figure 5E, E′, F, K, K′ and L) or RING-SH2^Drk^ (Figure 5 C, C′, D, I, I′ and J), compared to those observed in wild-type cells (Figure 5A, A′, B, G, G′ and H). The frequencies of detecting co-localization signals of HRP-Grk and Rab5-GFP/Rab7-GFP were 17%/15% (n = 14/n = 12 egg chambers) in wild-type, 35%/26% (n = 8/n = 22) and 38%/23% (n = 16/n = 14) in cells expressing D-CblL and RING-SH2^Drk^, respectively. Furthermore, the signals of exogenous D-CblL or RING-SH2^Drk^ proteins detected by anti-Flag antibody were trapped near the cell cortex in follicle cells expressing dominant negative Rab5S43N (data not shown). We then further examined the multivesicular body (MVB) sorting of D-CblL- and RING-SH2^Drk^-mediated trafficking using the anti-Hrs antibody that labels sorting endosomes. 22% (n = 12) and 29% (n = 11) of HRP-Grk signals were co-colocalized with Hrs signal in follicle cells respectively expressing D-CblL (Figure 5 Q, Q′ and R) or RING-SH2^Drk^ (Figure 5 O, O′ and P), whereas only 16% (n = 12) HRP-Grk/Hrs co-localization signal were observed in wild-type egg chambers (Figure 5 M, M′ and N). This observation suggests that RING-SH2^Drk^-mediated endocytosis shares the same trafficking route with D-CblL through the early endosome, late endosome and MVB to lysosome, and all steps of endocytosis are increased in the D-CblL or RING-SH2^Drk^ expressing cells.

**Figure 6:**
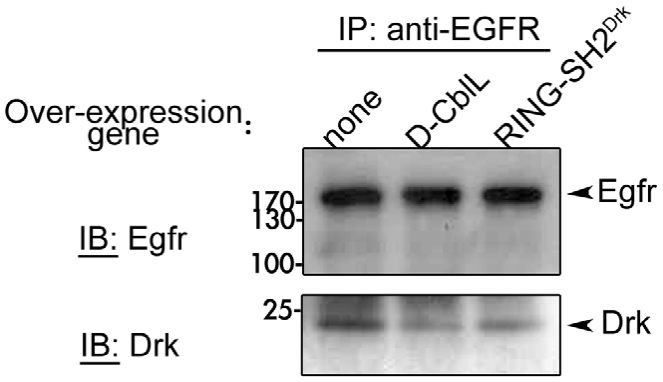
The amount of Drk in the EGFR complex. The EGFR complex were immunoprecipitated by mouse monoclonal anti-EGFR anti-bodies from ovariant lysates extracted from wild-type females or females expressing D-CblL, or RING-SH2^Drk^ in their follicle cells. The immunoprecipitate was separated by SDS-PAGE, and analyzed by rabbit polyclonal anti-EGFR (upper panel) or anti-Drk antibodies (lower panel). The EGFR signal appeared near 170kD (arrowhead in upper panel), and the Drk signal was near 24kD (arrowhead in lower panel).

Because the SH2 domain of RING-SH2^Drk^ was derived from Drk, we assumed that the docking site for RING-SH2^Drk^ on EGFR is the same as that for Drk. Therefore, this chimeric protein might compete with endogenous Drk for binding to EGFR. This possibility was tested by immunoprecipitation using anti-EGFR antibodies to determine the amount of Drk in the receptor complex. 40% of Drk in the EGFR complex was reduced in egg chambers expressing RING-SH2^Drk^ or full length D-CblL, compared to the wild-type egg chambers (Figure 6). This result indicates that RING-SH2^Drk^ interferes with the interaction between endogenous Drk and EGFR, which may lead to reduced signal transduction. Taken together, the chimeric protein RING-SH2^Drk^ may down-regulate EGFR signaling through promoting the endosomal trafficking of the EGFR complex and reducing the recruitment of Drk/Sos in signal transduction.

## Discussion

To dissect the molecular machinery for EGFR endocytosis, we studied the mechanism by which D-CblL promotes EGFR endocytosis in *Drosophila* follicle cells. We then applied our results to generate a chimeric protein, containing the RING finger domain of Cbl fused to the SH2 domain of Drk, which can very effectively down-regulate EGFR signaling.

### Drk plays two roles in EGFR signaling in *Drosophila* oogenesis

Mammalian studies have reported that c-Cbl regulates EGFR signaling through its interacting molecules, such as Grb2, CIN85-Endophilin complex and Eps15 [21]. This study investigated the role of these molecules in D-CblL mediated regulation, and found that elimination of Drk interaction resulted in significant reduction of the effect of D-CblL over-expression (Table 1). In consideration of our data and results from previous studies, we conclude that the Grb2/Drk has dual roles in EGFR signaling both in mammals and *Drosophila*. Acting as a signaling transducer, Grb2 binds to the proline rich motif of RasGEF/Sos through the SH3 domain, leading to Ras activation and activation of MAPK cascade [46]. Similarly, loss of function of a *drk* mutant in *Drosophila* caused a ventralization of the egg, a phenotype representing hypo-activation of EGFR signaling [47]. In contrast, a recombinant SH2 domain of Grb2 inhibited EGFR endocytosis, indicating the requirement of Grb2 in EGFR endocytosis [48]. Here, we demonstrated that interaction between D-CblL and Drk is crucial for promoting EGFR endocytosis by D-CblL. Our protein interaction data in this study (Figure 1F and Figure S2) agree with previous finding that Drk/Grb2 interacts with the proline-rich motif of Cbl [49], [50]. In cell culture studies, the indirect binding of EGFR to Cbl through Grb2 is necessary for receptor internalization, whereas endosomal sorting requires direct binding to Cbl [31], [35]. Our findings showed that the chimeric protein is sufficient to down-regulate EGFR signaling and suppresses the effect of λ-top over-expression in *Drosophila* ovaries (Table 3 and 4). Expression of RING-SH2^Drk^ facilitated the endosomal trafficking of ligand-receptor complex through Rab5 (early) and Rab7 (late) endosomes, and MVBs (Figure 5). This result argues that recruiting the RING domain of Cbl to EGFR by the SH2 domain of Drk can promote the trafficking of EGFR to degradation compartments, such as MVBs. Furthermore, expression of RING-SH2^Drk^ led to reduced Drk binding on EGFR (Figure 6). This observation suggests that RING-SH2^Drk^ chimeric protein not only promotes the trafficking of EGFR to lysosomal degradation pathway, but also competes away the endogenous Drk, which may also contribute to reducing EGFR signaling. Interestingly, over-expression of D-CblL also reduced the binding of Drk to EGFR, suggesting that D-CblL might sequester Drk from binding to the receptor for signaling.

**Table 4:** D-CblL, RING-SH2^Drk^ and RING-PR suppresses *λ-top* effects.

	% Phenotype of eggshell					
Genotype¶	V3/V2*	V1*	Wt*	WtD*	D1/D2*	N
*λ-top^38B^; LacZ*	0	0	52	39	9/0	273
*D-CblL-A12*	86	14	0	0	0/0	228
*λ-top^38B^; D-CblL-A12*	9	34	56	1	0/0	165
*RING-SH2^Drk^*	41	44	15	0	0/0	228
*λ-top^38B^; RING-SH2^Drk^-2*	0	0	95	5	0/0	219
*λ-top^4.2^; LacZ*	0	0	0	7	0/93	210
*λ-top^4.2^; RING-PR-A, RING-PR-D*	0	0	6	19	9/66	227

*The eggshell pattern of Wt, V1, V2 and V3 were the same as that described in Table1. WtD indicated extra dorsal appendage appeared in the dorsa-lateral region; D1 indicated two dorsal appendages appeared in lateral region, and the dorsal midline area was expanded. D2 indicated the dorsalized eggshell with dorsal appendage around anterior of egg.

¶ The egg collection was done at 29°C, and expressions were driven by *EQ1-Gal4*.

Importantly, the lethal effect of D-CblL was significantly reduced when the Drk binding motif was mutated in D-CblL (Table 2). This indicates that D-CblL efficiently down-regulates EGFR signaling through Drk. Large amounts of D-CblL in the cell may lead to comprised EGFR signaling levels that are too low for survival. However, even when we eliminated the interaction with Drk in the D-CblL-mPR mutant, this protein could still down-regulate λ-top (Table S3). This result further demonstrated that D-CblL down-regulates EGFR through multiple mechanisms, and other D-CblL interacting molecules besides Drk might play important roles in down-regulating EGFR signaling even when Drk is absent.

### The role of the RING finger domain in Cbl-mediated down-regulation of EGFR signaling

Our previous study demonstrated that *D-cbl* is required for down-regulation of EGFR signaling during DV patterning of the eggshell and embryo. Here we further showed that the RING finger domain of D-CblL is essential for its negative effect on EGFR, since the D-CblL mutant protein lacking this domain (Δ70Z-D-CblL) exhibited a dominant negative effect (Figure 2). In addition, the Δ70Z-D-CblL mutant also failed to rescue *cbl^F165^* mutant, indicating that RING finger domain activity plays a major role in D-CblL function. These data are consistent with results from studies on the D-Cbl loss-of-function alleles by Wang et al. [13] and from research reports in mammals [42], [51], [52]. Ubiquitination has been considered as a signal to mediate EGFR endocytosis at two critical steps: receptor internalization and endosormal sorting [17], [31], [35], [53], [54]. D-CblL promotes EGFR endocytosis in a dose-dependent manner, but the endocytosis of the ligand-receptor complex was significantly reduced in Δ70Z-D-CblL over-expressing follicle cells. Interestingly, a cell culture system that expressed an EGFR mutant with a reduced ubiquitination level (to only 1%) still displayed normal internalization [53]. Therefore, one possibility is that the endocytic signal is not ubiquitination of EGFR itself [53], and that other molecules might be involved and ubiquitinated by Cbl. Cbl can directly bind the Y1045 residue of EGFR through its SH2 domain. However, the binding-deficient Y1045F EGFR mutant is internalized almost as efficiently as the wild-type EGFR, indicating that direct binding of Cbl to EGFR is not necessary for EGFR endocytosis [55]. Indeed, the chimeric protein (RING-SH2^Drk^) we generated promoted ligand-receptors endocytosis as well (Figure 4 and Figure 5). Therefore, we conclude that direct interaction between Cbl and EGFR may not be necessary for ligand-receptor endocytosis in vertebrate and invertebrate cells. Considering that Cbl acts as an adaptor in many signaling pathways [21], we were surprised to find that the RING finger domain, while recruited specifically to EGFR, is sufficient to down-regulate EGFR signaling. This finding implies the possibility of using the RING finger domain as a therapeutic tool in human diseases treatment.

## Materials and Methods

### Fly strains

The strains used include: wild-type (*Oregon-R, OreR*); *Drk^EOA^/CyO* [39]; *Eps15^e75^* and *Eps15^Δ25^* [56]; *endoB^54^/CyO* (Pai unpublished research); *UAS-CblL-A9*; *UAS-CblL-A10*; *UAS-CblL-A12*; *UAS-CblL-A18; Hs83-CblL-6* and *Hs83-CblS-2* [30]; *EQ1* and *GR1* Gal4 lines [57]; *cbl^F165^/TM3* [11]; *UAS-λ-top* [57]; *UAS-GFP-Rab5* [58]; *UAS-GFP-Rab7* [59]; *HRP-grk-5* [29].

### DNA constructs

To generate Flag-D-CblL, the D-CblL 328-3533 cDNA fragment was cloned into pUAST-Flag vector with *Bag*II/*Kpn*I sites. D-CblL-Δ70Z was generated by site-directed mutagenesis, which was designed according to the previously reported method [20]. To generate the D-CblL-mPR^Drk^ mutant, we replaced the Pro^475^ and Pro^479^ with Ala using site-directed mutagenesis. The primers used include: Cbl-mut-2270-F 5′-gtggtggctgctcccctgccagcccgaaagtcctcacc-3′ and Cbl-mut-2270-R 5′-ggtgaggactttcgggc tggcaggggagcagccaccac-3. To generate pUAST-Flag-RING, the RING finger domain of D-CblL was amplified by PCR using the following primers: RING-F-KpnI 5′-aaggtaccggaccac ataaccgttacccaagag-3′ and RING-R-EcoRI 5′-aagaattctcagtgtcgtcttcttcc-3′. The RING fragment was cloned into pUAST-Flag using *Kpn*I and *EcoR*I sties and was in-frame fused to 3′ of the Flag-tag. To generate pUAST-Flag-RING-SH2^Drk^, the SH2 domain of Drk (amino acid 53 to 160) was amplified from the *Drosophila* ovarian cDNA library by PCR using the following primers: SH2-F-RI 5′-aagaattcaatagaaatgaagaatcacgactggtat-3′ and SH2-R-XbaI 5′-aatctagacagcgcctg cacgag-3′. The SH2^Drk^ fragment was cloned into pUAST-Flag-RING using *EcoR*I and *Xba*I sties and was in-frame fused to 3′ of the RING finger domain. To generate the pUAST-Flag-RING-PR, the following two poly-nucleotides PR-F 5′-aattcacctcccctgccaccccgat-3′ and PR-R 5′-ctagatcggggtggcaggggaggtg-3′ were synthesized in vitro, and they were ligated to the pUAST-Flag-RING. Therefore, the PR motif is in-frame fused with the RING domain.

### Antibodies

The antibodies used for immunocytochemistry were the mouse monoclonal anti-Gurken antibody 1D12 [60]; the goat anti-HRP antibody (Jackson ImmunoResearch, 1∶250 dilution); the mouse anti-Flag (Sigma, 1∶250 dilution); the guinea pig anti-FL-Hrs [61]; the Alexa Fluor 499 or 546 goat anti-mouse antibody (Molecular Probes); the Alexa Fluor 488 or 546 rabbit anti-goat antibody (Molecular Probes); the TRITC-conjugated donkey anti-mouse antibody (Jackson ImmunoResearch); and the FITC-conjugated donkey anti-guinea pig antibody (Upstate). The following antibodies were used for immunoblotting: mouse anti-D-CblL 8C4 ascites [30], mouse anti-D-Cbl 10F1 ascites [30], mouse anti-Flag (sigma, 1∶500 dilution), rabbit anti-Drk [39], mouse anti-tubulin (Sigma, 1∶3000 dilution).

### Immunoprecipitation

The 70 pairs of ovaries were dissected and homogenized with 400 ml lysis buffer (50 mM HEPES, 50 mM NaCl, 10% glycerol, 1% Triton-X, 1 mM EGTA, 1 mM EDTA, 1 mM NaVO_3_, 1 mM DTT, 10 mM NaF, 10 mM N-ethylmleimide, 0.5% sodium deoxycholate). The lysates were sonicated and separated the supernatants by centrifugation at 8000 g for 10 minutes at 4°C. The supernatants were incubated with anti-D-Cbl antibodies rE6 (1∶100) or mouse anti-D-Egfr (1∶50 Sigma) for 1.5 hours at 4°C. Then protein A sepharose beads (for rabbits anti-body) or protein G sepharose beads (for mouse anti-body) were added to bring down the immunoprecipitates for 2 hours at 4°C. Pellets were dissolved in 30 µl 2x sample buffer, boiled for 10 minutes. The samples 10 µl of immunoprecipitates or 30 µl of total lysate were separated by SDS-PAGE and detected with anti-Drk [39], 8C4 [30], rabbit anti-EGFR [29] antibodies.

### Yeast two-hybrid analyses

The full-length Drk was amplified by PCR from the *Drosophila* ovarian cDNA library and cloned into the yeast expression plasmid pGBDT7 (Clontech) to fuse with the DNA-binding domain of GAL4 protein. The pGBKT7 vector was the negative control bait. The proline-rich domain of D-CblL was amplified by PCR and cloned into the yeast expression plasmid pGADT7 to fuse with the activation domain of GAL4 protein. The bait vector carrying the *TRP1* gene and the prey vector carrying the *LEU2* gene were for auxotrophic selection. The pCL1 plasmid carrying full length GAL4 protein was used as a positive control. The pGBDT7-Drk was co-transformed into an YH109 yeast strain (Clontech) either with pGADT7-mPR, or pGADT7-wild-type-PR or pGADT7 vector, and the transformants were grown on leucine-tryptophan double selective plates.

## Supporting Information

Figure S1 **The expression levels of transgenes.** (A) To compare the expression levels between D-CblL and D-CblL-mPR transgenic lines, *UAS-D-cblL* (A10 and A12) and *UAS-D-cblL-mPR* mutants (AE and GB) were expressed in follicle cells driven by *EQ1-Gal4* at 25°C. The endogenous D-CblL level was detected in *OreR* ovary extract. LE indicated long exposure, and SE indicated short exposure. (B) To compare the expression level of each *hs83-D-cblL* transgenic line, the protein samples were extracted from ovaries carrying the *hs83-D-CblL* or *hs83-D-CblL-mPR* mutant gene. The D-CblL and D-CblL-mPR were detected by a mouse monoclonal anti-body (8C4). (C) To compare the expression level of each Flag-D-CblL and Flag-RING-SH2^Drk^ line, the full-length D-CblL and chimeras' expression levels were analyzed by the anti-Flag anti-body. The transgenic lines used are as follows: Flag-L1, Flag-L6, Flag-RING-SH2^Drk^-2 and Flag-RING-SH2^Drk^-5. (D) To compare the expression level of RING, RING-PR and RING-SH2^Drk^ transgenic lines, the RING, RING-PR or RING-SH2^Drk^ was expressed by *GR1-Gal4* at 29°C (left panel). We expressed two copies of RING or RING-PR either by *GR1-Gal4* at 32°C or a stronger driver, *hsGal4*, at 37°C (right panel). The ovarian protein extracts were separated by SDS-PAGE and analyzed by anti-Flag anti-body. The number indicated expression level of each line, and the tubulin served as a loading control. Flag-RING is about 13 kD. Flag-RING-PR is about 14kD, and Flag-RING-SH2^Drk^ is about 25kD.
(TIF)

Figure S2 **Drk interacts with the PR motif of D-CblL.** (A) The interaction between Drk and D-CblL was analyzed by a yeast-two hybrid system. Yeasts are co-transformed with pGFBKT7-Drk-FL and pCL1 (as a positive control), pGADT7 vector (as a negative control), wild-type or mPR proline-rich domain. (B) On the G2 selection plate, the duplicated experiments show the successful transformation of each line. (C) On the G3 selection plate, yeast containing the PR mutant or the pGADT7 vector could not grow, whereas yeast containing the wild-type or pCL1 plasmid could grow.
(TIF)

Table S1 Over-expression of D-CblL has little genetic interaction with *D-eps15* and *D-endophilin B*.
(DOC)

Table S2 The effect of D-CblL over-expression is suppressed in *eff^8^* heterozygote mutant.
(DOC)

Table S3 D-CblL-mPR partially suppresses *λ-top* effects.
(DOC)
