# Supplementary figures and images for: D-Cbl Binding to Drk Leads to Dose-Dependent Down-Regulation of EGFR Signaling and Increases Receptor-Ligand Endocytosis
Source: PLoS One. 2011 Feb 14;6(2):e17097. doi: 10.1371/journal.pone.0017097 (PMC3038869; doi:10.1371/journal.pone.0017097)

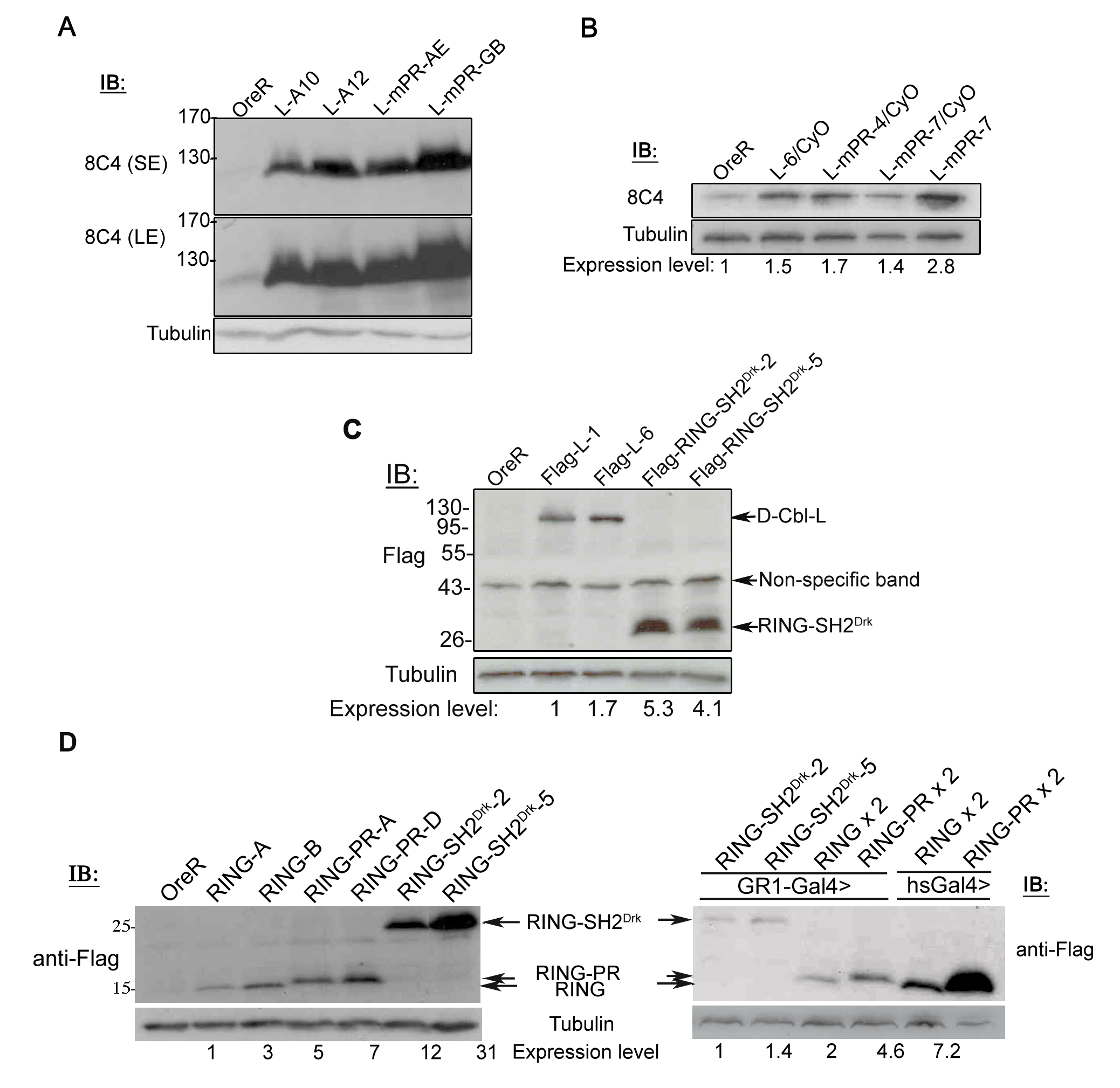

Supplement: Figure S1 — The expression levels of transgenes. (A) To compare the expression levels between D-CblL and D-CblL-mPR transgenic lines, UAS-D-cblL (A10 and A12) and UAS-D-cblL-mPR mutants (AE and GB) were expressed in follicle cells driven by EQ1-Gal4 at 25°C. The endogenous D-CblL level was detected in OreR ovary extract. LE indicated long exposure, and SE indicated short exposure. (B) To compare the expression level of each hs83-D-cblL transgenic line, the protein samples were extracted from ovaries carrying the hs83-D-CblL or hs83-D-CblL-mPR mutant gene. The D-CblL and D-CblL-mPR were detected by a mouse monoclonal anti-body (8C4). (C) To compare the expression level of each Flag-D-CblL and Flag-RING-SH2Drk line, the full-length D-CblL and chimeras' expression levels were analyzed by the anti-Flag anti-body. The transgenic lines used are as follows: Flag-L1, Flag-L6, Flag-RING-SH2Drk-2 and Flag-RING-SH2Drk-5. (D) To compare the expression level of RING, RING-PR and RING-SH2Drk transgenic lines, the RING, RING-PR or RING-SH2Drk was expressed by GR1-Gal4 at 29°C (left panel). We expressed two copies of RING or RING-PR either by GR1-Gal4 at 32°C or a stronger driver, hsGal4, at 37°C (right panel). The ovarian protein extracts were separated by SDS-PAGE and analyzed by anti-Flag anti-body. The number indicated expression level of each line, and the tubulin served as a loading control. Flag-RING is about 13 kD. Flag-RING-PR is about 14kD, and Flag-RING-SH2Drk is about 25kD. (TIF) [file pone.0017097.s001.tif]

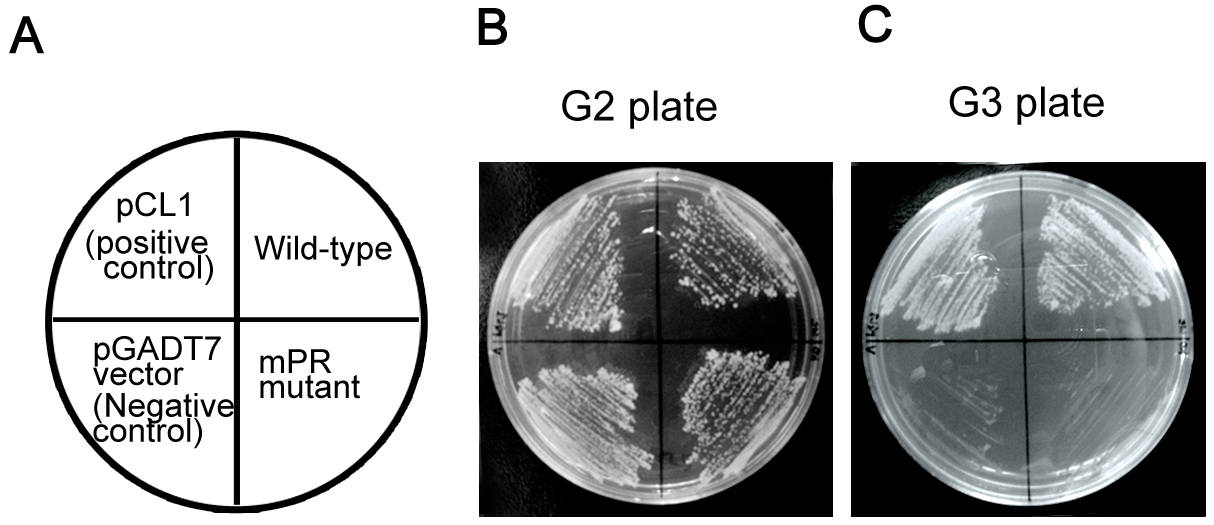

Supplement: Figure S2 — Drk interacts with the PR motif of D-CblL. (A) The interaction between Drk and D-CblL was analyzed by a yeast-two hybrid system. Yeasts are co-transformed with pGFBKT7-Drk-FL and pCL1 (as a positive control), pGADT7 vector (as a negative control), wild-type or mPR proline-rich domain. (B) On the G2 selection plate, the duplicated experiments show the successful transformation of each line. (C) On the G3 selection plate, yeast containing the PR mutant or the pGADT7 vector could not grow, whereas yeast containing the wild-type or pCL1 plasmid could grow. (TIF) [file pone.0017097.s002.tif]
