# Supplementary material for: D-Cbl Binding to Drk Leads to Dose-Dependent Down-Regulation of EGFR Signaling and Increases Receptor-Ligand Endocytosis
Source: PLoS One. 2011 Feb 14;6(2):e17097. doi: 10.1371/journal.pone.0017097 (PMC3038869; doi:10.1371/journal.pone.0017097)
Supplement: Table S1 — Over-expression of D-CblL has little genetic interaction with D-eps15 and D-endophilin B. (DOC) [file pone.0017097.s003.doc]

**Table S1** Over-expression of D-CblL has little genetic interaction with *D-eps15* and *D-endophilin B*.

| **At 25°C** | **% Eggshell phenotype** | | | |  |
| --- | --- | --- | --- | --- | --- |
| **The genotypes of females** | **V3** | **V2** | **V1** | **Wt** | **N** |
| *EQ1>D-cblL-A12* | 8 | 76 | 16 | 0 | 278 |
| *endoB^54^/+^¥^* | 0 | 0 | 0 | 100 | 179 |
| *eps15^EP2513^/+* | 0 | 0 | 0 | 100 | 197 |
| *EQ1>D-cblL-A12 in endoB^54^/+*^¥^ | 0 | 66 | 34 | 0 | 149 |
| *EQ1>D-cblL-A12 in eps15^e75^/+* | 0 | 83 | 16 | 1 | 291 |
| *EQ1>D-cblL-A12 in eps15^EP2513^/+* | 0 | 82 | 18 | 0 | 229 |

^¥^  *endoB^54^* is a protein null mutant, generated by P-element excision (data not shown).
