# Supplementary material for: D-Cbl Binding to Drk Leads to Dose-Dependent Down-Regulation of EGFR Signaling and Increases Receptor-Ligand Endocytosis
Source: PLoS One. 2011 Feb 14;6(2):e17097. doi: 10.1371/journal.pone.0017097 (PMC3038869; doi:10.1371/journal.pone.0017097)
Supplement: Table S2 — The effect of D-CblL over-expression is suppressed in eff8 heterozygote mutant. (DOC) [file pone.0017097.s004.doc]

**Table S2** The effect of D-CblL over-expression is suppressed in *eff^8^* heterozygote mutant.

| **At 25°C** | **% Eggshell phenotype** | | | |  |
| --- | --- | --- | --- | --- | --- |
| **The genotypes of females** | **V3** | **V2** | **V1** | **Wt** | **N** |
| *EQ1>D-cblL-A18* | 36 | 62 | 2 | 0 | 292 |
| *EQ1>D-cblL-A18* in *eff^8^/+* | 0 | 74 | 25 | 1 | 262 |
