# Supplementary material for: D-Cbl Binding to Drk Leads to Dose-Dependent Down-Regulation of EGFR Signaling and Increases Receptor-Ligand Endocytosis
Source: PLoS One. 2011 Feb 14;6(2):e17097. doi: 10.1371/journal.pone.0017097 (PMC3038869; doi:10.1371/journal.pone.0017097)
Supplement: Table S3 — D-CblL-mPR partially suppresses λ-top effects. (DOC) [file pone.0017097.s005.doc]

**Table S3** D-CblL-mPR partially suppresses *λ-top* effects.

| **Driven at 29°C by *EQ1-Gal4*** | **% Phenotype of eggshell** | | | |  |
| --- | --- | --- | --- | --- | --- |
| **Genotype** | **V3/ V2** | **V1** | **Wt** | **WtD** | **N** |
| *λ-top^38B^; LacZ* | 0 | 0 | 4 | 96 | 432 |
| *λ-top^38B^; D-CblL-A12* | 28.2 | 67.3 | 4.5 | 0 | 269 |
| *λ-top^38B^; D-CblL-mPR-GB* | 11 | 0 | 51 | 38 | 229 |
